# Attention deficit/hyperactivity disorders with co-existing substance use disorder is characterized by early antisocial behaviour and poor cognitive skills

**DOI:** 10.1186/1471-244X-13-336

**Published:** 2013-12-13

**Authors:** Berit Bihlar Muld, Jussi Jokinen, Sven Bölte, Tatja Hirvikoski

**Affiliations:** 1 SiS LVM Institution Hornö, Enköping, Sweden; 2 Department of Clinical Neuroscience, Karolinska Institutet, Stockholm, Sweden; 3 Department of Women’s and Children’s Health, Karolinska Institute, Pediatric Neuropsychiatry Unit, Center of Neurodevelopmental Disorders at Karolinska Institutet (KIND), Stockholm, Gävlegatan 22B, SE-113 30, Sweden

**Keywords:** ADHD, SUD, Co-morbidity, Compulsory care, Adults, Cognition

## Abstract

**Background:**

Attention Deficit/Hyperactivity Disorder (ADHD) is associated with an increased risk of co-existing substance abuse. The Swedish legislation on compulsory healthcare can be applied to persons with severe substance abuse who can be treated involuntarily during a period of six months. This context enables a reliable clinical assessment of ADHD in individuals with severe substance use disorder (SUD).

**Methods:**

In the context of compulsory care for individuals with severe SUD, male patients were assessed for ADHD, co-morbid psychiatric symptoms, psychosocial background, treatment history, and cognition. The data from the ADHD/SUD group (n = 60) was compared with data from (1) a group of individuals with severe substance abuse without known ADHD (SUD group, n = 120), as well as (2) a group with ADHD from an outpatient psychiatric clinic (ADHD/Psych group, n = 107).

**Results:**

Compared to the general SUD group in compulsory care, the ADHD/SUD group had already been significantly more often in compulsory care during childhood or adolescence, as well as imprisoned more often as adults. The most common preferred abused substance in the ADHD/SUD group was stimulant drugs, while alcohol and benzodiazepine abuse was more usual in the general SUD group. Compared to the ADHD/Psych group, the ADHD/SUD group reported more ADHD symptoms during childhood and performed poorer on all tests of general intellectual ability and executive functions.

**Conclusions:**

The clinical characteristics of the ADHD/SUD group differed from those of both the SUD group and the ADHD/Psych group in several respects, indicating that ADHD in combination with SUD is a particularly disabling condition. The combination of severe substance abuse, poor general cognitive ability, severe psychosocial problems, including indications of antisocial behaviour, and other co-existing psychiatric conditions should be considered in treatment planning for adults with ADHD and SUD.

## Background

Attention-Deficit/Hyperactivity Disorder (ADHD) is an early-onset neurodevelopmental disorder characterized by deficiencies in impulse control, regulation of activity level, and attention. ADHD also frequently involves executive dysfunctions, such as problems of planning, organization, getting started, and completing activities. While the prevalence of ADHD in the population is about 1.2 – 7.3% [1], a recent meta-analysis estimated the prevalence to be 19–27% in substance abusers, depending on which preferred substance is studied [2]. A study in a high-security prison in Sweden estimated the prevalence of adult ADHD among longer-term inmates to be 40%, and the majority of clinically assessed individuals with ADHD also had previous SUD [3]. Furthermore, a 10-year follow-up study of children and adolescents with ADHD found that the condition is a significant predictor of any type of substance use disorder (SUD) [4].

The increased risk for developing substance abuse among individuals with ADHD may be related to the core symptoms of ADHD, such as impulsivity, as well as associated problems in ADHD, such as weakness in executive regulation of affects and motivation. Adults with ADHD also report high levels of stress in everyday life and poor coping ability [5] and substance abuse may be a destructive coping strategy. Psychiatric co-morbidity is common in clinically referred adults with ADHD. Eighty % present with at least one additional DSM-IV diagnosis [6-8] and co-morbidity as such may also increase the risk of substance abuse. Furthermore, psychosocial factors such as failure in school and working life and experiences of alienation among individuals with ADHD may combine to increase the risk of substance abuse. A low dopamine activity in persons with ADHD and the capacity of all addictive substances to increase the release of dopamine has been suggested to be a possible neural link between ADHD and SUD [9,10].

The Law on Care of Alcoholics and Drug Abusers (LVM) is one of four laws governing involuntary care in Sweden. The National Board of Institutional Care (SiS) is the authority responsible for compulsory treatment of adults with severe substance abuse, according to the LVM, as well as for compulsory care of children and adolescents with severe social problems such as substance abuse and antisocial behaviour, according to the law on Care of Young Persons (LVU).

In Sweden, approximately 1000 individuals per year are sentenced to compulsory care for severe substance abuse in accordance with the LVM. There are more than 300 treatment sites that are distributed among 11 institutions around the country. In recent years, assessments of neurodevelopmental disorders have frequently been requested during the compulsory care under LVM. In many cases, it has not been possible to perform this type of assessment due to an on-going substance abuse.

In the scientific literature, adults with ADHD have been clinically characterized in psychiatric and forensic [3] settings. However, to the best of our knowledge, there are no studies describing the clinical characteristics of adults with ADHD and severe substance abuse in compulsory care.

The objective of the present study is a clinical characterization of patients with ADHD and severe substance abuse disorder in a compulsory care context. Our aim is to increase the clinical understanding of this patient group in order to facilitate adjustment of treatment for this clinically challenging group. In order to characterize this group, a comparison has been made with two other groups: a group of patients with SUD in compulsory care without a known ADHD and a group of individuals with ADHD from a psychiatric outpatient setting.

## Methods

### Study settings

The assessment of the study group was made between the years 2004 and 2008 at an SiS institution, SiS LVM Institution Hornö, located in Enköping, Sweden. The patient target of the SiS Institution Hornö is adult males who, in addition to substance abuse, are violent and/or may have other severe psychiatric co-morbidity. The patients come from different municipalities in Sweden and have been placed at an institution of a central unit of the National Board of Institutional Care. The SiS LVM Institution Hornö has conducted clinical assessments for many years in order to obtain data for individualized treatment planning, including treatment for ADHD. The voluntary assessments are conducted when requested by the patient’s social worker, the patient himself or the ward staff.

### Participants

**Figure 1:**
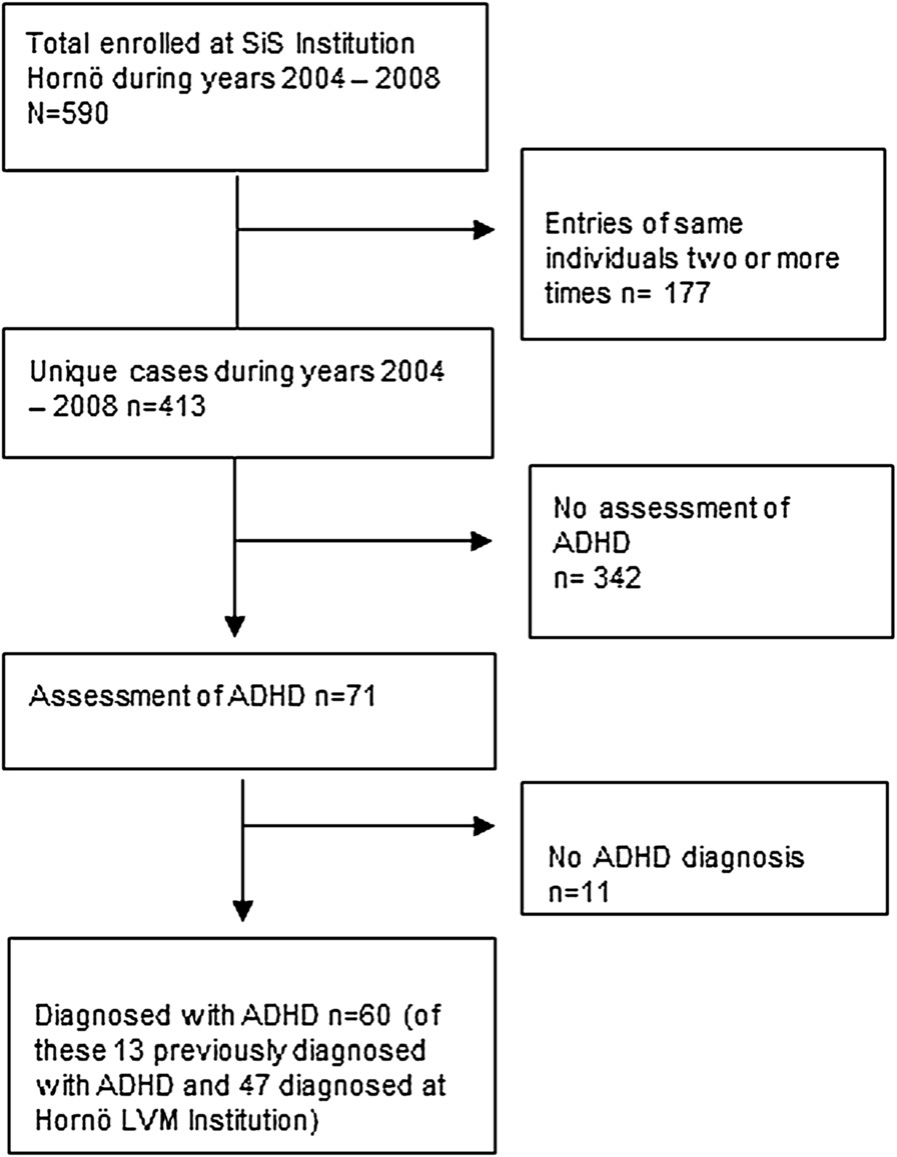
Flowchart describing enrolment of participants in the ADHD/SUD group from the SiS institution Hornö.

Figure 1 displays a flowchart describing the enrolment of participants in the ADHD/SUD group. Between the years 2004 and 2008, there were 413 individuals who were treated at the SiS Institution Hornö. During this period a total of 214 psychological assessments were made with 71 patients being assessed for ADHD. The participants in the ADHD/SUD group consist of 60 patients diagnosed with ADHD and recruited consecutively from the SiS Institution Hornö. Thirteen of these 60 clients had received an ADHD diagnosis prior to the involuntary care at Hornö Institution. The mean age of the participants was 26. 33 (SD = 6.07) years at admission to the Institution and 27.08 (SD = 6.02) at the beginning of the diagnostic assessment of ADHD (see below), with a range of 20 to 46 years for both mean age figures.

In order to characterize the ADHD/SUD group in compulsory care, we used two control groups. First, a general SUD group in compulsory care without known ADHD diagnosis and second, a group with ADHD without severe SUD in voluntary outpatient psychiatric care (ADHD/Psych group). Data for these control groups were extracted as follow.

In order to compare the characteristics of the combined ADHD/SUD group with the general SUD population in involuntary care, we used unidentified data from the SiS internal database (DOK, a SiS follow-up and documentation system), matched for age and years of assessment and covering the majority of the treated patients. During the time period for current study (2004–2008), a total of 1968 male clients were registered in DOK database. We selected data on 120 male clients without known ADHD diagnosis matched with the ADHD/SUD group from Hornö Institution for age and year of admission.

For comparison of the ADHD/SUD group the SiS Institution from Hornö with individuals with ADHD in voluntary psychiatric outpatient care, we used data on patients assessed at an outpatient tertiary psychiatric clinic, the Neuropsychiatric Unit Karolinska, Psychiatry Northwest, Stockholm, Sweden. The data on the ADHD/Psych group were extracted from an anonymous database, and the inclusion/matching criteria were sex (males), age range (19 to 46 years), and year of diagnostic assessment (2004–2008). One hundred seven individuals met these criteria and were all included in the ADHD/Psych group. The ADHD/Psych group had a mean age of 29.6 years (SD = 7.28) and were thus somewhat older than the ADHD/SUD group (M =27.08, SD = 6.02). The difference in age was statistically significant, as indicated by Student’s t-test (*p* = .002).

Diagnoses of other neurodevelopmental disorders in addition to ADHD were obtained from the patient’s medical records. In addition to ADHD, 30.0% of the ADHD/SUD group and 40.2% of the ADHD/Psych group had a co-existing Asperger syndrome diagnosis or other neurodevelopmental disorders (dyslexia, dyscalculia, developmental coordination disorder, Tourette syndrome, pervasive developmental disorder or mental retardation).

None of the study groups (ADHD/SUD; general SUD; ADHD/Psych) were originally recruited to a research study but the assessments were performed as an ordinary clinical work. Thus, the study had a retrospective design and the participants did not give their written informed consents. The study was approved by the Regional Ethics Committee of Stockholm (2009/823-31/4 and 42-790-2012).

### Procedure

#### Assessment of background information

In compulsory care for SUD, interviews with the clients are conducted on admission, discharge and one year after discharge. The semi-structured interviews, which are voluntary, are conducted by treatment staff at the SiS institutions for compulsory treatment of severe SUD. The data from the interviews are stored in the SiS evaluation and documentation system, the DOK database, which provides a large amount of information intended to guide treatment planning, as well as to provide a basis for research, operational planning, and follow-up.

Not all clients in the study group had had the DOK interviews and, in these cases, the background data were studied during the clinical ADHD assessment (see below).

The assessment of the general SUD group without a known ADHD (n = 120) was obtained entirely from an anonymous database comprising all DOK-interviewed clients.

#### Assessment of ADHD

The assessments at the SiS Institution Hornö began when the clients were thoroughly detoxified, approximately two months after the initiation of treatment. Regular drug screening was general clinical practice throughout the treatment period at the Hornö Institution. The diagnostic assessment was made by clinical psychologists working at the institution and having solid professional experience in the field of neurodevelopmental disorders. The assessment was extensive, lasting approximately 20–25 hours in total for each individual and was based on multiple sources of information: a *semi-structured clinical interview* based on the Diagnostic and Statistical Manual of Mental disorders- Fourth Edition [11] was conducted in all cases. The clients also completed *standardized self-rating questionnaires* assessing childhood (WURS, Wender Utah Rating Scale) as well as adult (Brown ADD scale) ADHD-symptoms. When possible, collateral information (questionnaires and clinical interviews) was gathered from *the participants’ significant others* in order to obtain a more complete diagnostic history of each individual. When available, additional information was obtained from case files from child and adolescent psychiatry, from institutions for involuntary care during childhood and/or adolescence, as well as from adult psychiatry settings. The assessments also included *neuropsychological testing.* The diagnosis of ADHD was established after reaching a consensus between two or three clinical psychologists from the SiS LVM Institution Hornö, or between a psychologist from the institution and a consulting specialist in neuropsychology.

The diagnostic assessment of the ADHD/Psych group was made using similar procedures and methods, since this type of extensive multiple data-source, consensus-based diagnostic assessments were a standard in Sweden during that time period.

### Measures

The data in the comparisons of the ADHD/SUD group and the general SUD group were obtained from the evaluation and documentation system, DOK. In the ADHD/SUD group, 22 out of 60 never had a DOK interview and, in these cases, the data were obtained from the clinical assessments made somewhat later during the compulsory care period. The ADHD/SUD group was compared with the general SUD in compulsory care regarding psychosocial background and treatment history during childhood (background in the childhood family; psychiatric disorder and/or substance abuse in one or both parents; possible compulsory care during childhood and/or adolescence; educational support in primary school) and during adulthood (educational level; work experience; previous adult psychiatric care; previous imprisonment). Moreover, the ADHD/SUD group was compared with the SUD group regarding the preferred abuse substance and self-reported psychiatric symptoms.

The ADHD/SUD group was compared with the ADHD/Psych group regarding retrospective ADHD symptom reports during childhood, cognition, and co-morbidity with additional neurodevelopmental disorders. For retrospective assessment of childhood symptoms of ADHD, the Wender Utah Rating Scale (WURS) [12] was used. The WURS is a 61-item self-rating scale scored on a 5-point Likert scale, from 0 = not at all or slightly to 4 = very much. The WURS-25 includes twenty-five items (total score 0–100) that discriminate best between ADHD and controls. A cut-off score of 46 identifies 86% true positives and 99% true negatives of ADHD according to the instrument developers [12].

The general intellectual level (Full Scale IQ, FSIQ) was measured using the Wechsler Adult Intelligence Scale III (WAIS III) [13]. In addition to the FSIQ, the results of the WAIS-III can be divided into Verbal IQ (VIQ) and non-verbal or Performance IQ (PIQ). Moreover, data can be described on the level of four indexes: the Verbal Comprehensive Index (related to Verbal IQ) and the Perceptual Organization Index (related to the non-verbal IQ), as well as the two indexes loading on executive functions (EFs): the Working Memory Index and the Processing Speed Index [13].

### Statistical analyses

The group comparisons were made using the Chi-square test for categorical variables and Student’s t-test for continuous variables. In the t-tests, the degrees of freedom were corrected for unequal variance if indicated by Levene’s test for equality of variance. Effect sizes for t-tests were expressed as Cohen’s *d*[14] and interpreted as effect sizes of 0.2 to 0.3, a ‘small’ effect, around 0.5 (half an SD), a ‘medium’ effect, and ≥ 0.8, a ‘large’ effect. Effect sizes for Chi-square tests were expressed as *Φ* (Phi) and interpreted as a weak association (.10–.20), a moderate association (.20–.40), a relatively strong association (.40–.60), a strong association (.60–.80), or a very strong association (>.80).

In order to investigate whether the observed differences in WAIS indexes loading on EF were specific or explained by the general intellectual ability (FSIQ), we performed analyses of co-variance with the EF index as a dependent variable, with group (ADHD/SUD *versus* ADHD/Psych) as fixed factor while entering FSIQ as a co-variate. The alpha-level was set at .05, while .05 ≥ *p-values* ≥ .10 were regarded as statistical trends. The statistical analysis was performed using the SPSS statistical software package (IBM, SPSS™, version 20).

## Results

The flowchart describes the enrolment of participants in the ADHD/SUD group from the SiS Institution Hornö. Out of the 413 unique cases during 2004 and 2008, assessments of ADHD were made in 71 patients.

Regarding family background, educational and vocational history, treatment history, psychiatric symptoms, and the primary abused substance, the ADHD/SUD group (n = 60) was compared with the general SUD group (n = 120), matched for gender (all males), age and year of admission to compulsory care due to SUD.

### 1. Family background, education, and vocation history

The statistics from the analyses of the family background, education and vocation history are shown in Table 1.

**Table 1:** Family background, education, and work experience in the two groups in compulsory care due to substance abuse: ADHD/SUD group and the general SUD group without known ADHD

	**ADHD/SUD**	**General SUD**			
	**n = 60**	**n = 120**	***χ2***	***p***	***Φ***
**Family background**					
Parents separated before 7 years old	16 (26.7)	12 (10.0%)	7.77	.10	.21
Parents separated between ages 7 and 18	12 (20%)	23 (19.2%)			
Single parent	11 (18.3)	21 (17.5%)			
Other custodian than biological parents	4 (6.7)	7 (5.8%)			
Both parents	17 (28.3%)	46 (38.3%)			
Missing data	0	11 (9.2%)			
**Psychiatric disorder and/or abuse in parents**					
Psychiatric disorder and/ or abuse in parents	32 (53.4%)	68 (56.7%)	0.35	.85	.01
No psychiatric disorder and/or abuse in parents	26 (43.3%)	52 (43.3%)			
Missing data	2 (3.3%)	0			
**Educational level**					
Less than 9 years	12 (20.0%)	23 (19.2%)	2.35	.50	.11
Nine years	34 (56.7%)	58 (48.3%)			
Secondary school/ vocational education	14 (23.3%)	37 (30.8%)			
University/high school	0	2 (1.7%)			
**Work experience**					
Work experience ≤ 6 months	26 (43.3%)	40 (33.3%)	2.7	0.10	.12
Work experience > 6 months	30 (50.0%)	79 (65.9%)			
Missing data	4 (6.7%)	1 (0.8%)			

The ADHD/SUD group exhibited a trend towards a higher percentage of unstable family backgrounds compared to the general SUD group (*p* = .10). Separations in parents before seven years were more than twice as common in the ADHD/SUD group (27.7% versus 11.7%).

Psychiatric disorder and/or abuse in one or both parents were common in both groups (53.3% in the ADHD/SUD group and 56.7% in the general SUD group).

The educational level was low in both groups. In the ADHD/SUD group, 56.7% had completed the nine-year primary school and in the general SUD group, 48.3%.

The ADHD/SUD group showed a statistical trend towards less work experience than the general SUD group *(p* = .10).

### 2. Previous interventions and psychiatric care

The statistics from the analyses of previous treatment intervention and psychiatric care are shown in Table 2.

**Table 2:** Previous interventions and psychiatric care in the two groups in compulsory care due to substance abuse: ADHD/SUD group and the general SUD group without known ADHD

	**ADHD/SUD**	**General SUD**			
	**n = 60**	**n = 120**	***χ2***	***p***	***Φ***
**Special education in primary school**					
Special education in primary school	22 (36.7%)	N/A	N/A	N/A	N/A
No special education	36 (60%)	N/A			
Missing data	2 (3.3%)	N/A			
**Compulsory care during childhood (LVU)**					
LVU	26 (43.3%)	34 (28.4%)	4.24	**.04**	.15
Not LVU	33 (55.0%)	85 (70.8%)			
Missing data	1 (1.7%)	1 (0.8%)			
**Previous imprisonment**					
Imprisonment	41 (68.3%)	47 (39.2%)	15.8	**<.001**	.30
No imprisonment	16 (26.7%)	71 (59.1%)			
Missing data	3 (5.0%)	2 (1.7%)			
**Previous adult psychiatric care**					
Psychiatric care in adulthood	40 (66.7%)	65 (54.2%)	2.50	.12	.12
No psychiatric care in adulthood	18 (30.0%)	50 (41.6%)			
Missing data	2 (3.3%)	5 (4.2%)			

Note: N/A not assessed in general SUD group.

Note: P-values printed in bold indicate a statistically significant difference.

No data on special education in primary school in the general SUD group were available. In the ADHD group, the percentage of individuals who had received special educational support in primary school was 36.7%. Secondary school or vocational education was slightly more frequent in the general SUD group, 30.0%, compared to 23.3% in the ADHD/SUD group. The ADHD/SUD group had a significantly higher degree of compulsory care during childhood (LVU) than the general SUD group (*p* = .040). No data on the causes of compulsory care during childhood were available for the general SUD group, but in the ADHD/SUD group, the most dominant cause was early onset of substance abuse and antisocial behaviour.

Imprisonment was significantly more frequent in the ADHD/SUD group (68.0%), compared to the general SUD group (39.1%) (*χ*^*2*^ *=* 15.84, *p* < .001).

A small difference, approaching a statistical trend (*p* = .12), could be seen between the ADHD/SUD and the general SUD groups in previous experiences of psychiatric care (66.7% in the ADHD/SUD group versus 54.2% in the general SUD group).

### 3. Psychiatric symptoms and primary substance abuse

The statistics from the analyses of psychiatric symptoms and primary substance abuse are shown in Table 3.

**Table 3:** Psychiatric symptoms and primary substance abuse in the two groups in compulsory care due to substance abuse: ADHD/SUD group and the general SUD group without known ADHD

	**ADHD/SUD**	**General SUD**			
	**n = 60**	**n = 120**	***χ2***	***p***	***Φ***
**Self-reported hallucinations and other psychotic symptoms**					
Hallucinations and other symptoms of psychosis	35 (58.4%)	77 (64.1%)	.04	.84	.02
No hallucinations or other symptoms of psychosis	20 (33.3%)	41 (34.2%)			
Missing	5 (8.3%)	2 (1.7%)			
**Self-reported symptoms of depression and anxiety**					
Symptoms of depression/anxiety	51 (85.0%)	101 (84.2%)	.69	.41	.06
No symptoms of depression/anxiety	6 (10.0%)	18 (15.0%)			
Missing data	3 (5.0%)	1 (0.8%)			
**Preferred abused substance**					
Heroin	12 (20.0%)	25 (20.8%)	18.3	.**049**	.32
Amphetamine	29 (48.3%)	36 (30.0%)			
Cocaine	3 (5.0%)	1 (0.8%)			
Alcohol	4 (6.7%)	26 (21.7%)			
Hashish/marijuana	7 (11.7%)	11 (9.2%)			
GHB	3 (5.0%)	3 (2.5%)			
Benzodiazepines	2 (3.3%)	9 (7.5%)			
Opiates other than heroin, including analgesics	0	3 (2.5%)			
Solvents	0	3 (2.5%)			
Other drugs	0	2 (1.7%)			
Missing data	0	1 (0.8%)			
**Abuse according to drug screening (not alcohol)**					
Benzodiazepines	35 (58.3%)	N/A			
Hashish/marijuana	29 (48.3%)	N/A			
Amphetamine	33 (55.0%)	N/A			
Opiates/Substitution drugs	17 (28.3%)	N/A	N/A	N/A	N/A
LSD/GHB/Ecstasy	6 (10.0%)	N/A			
Missing data due to detoxification before admission	10 (16.7%)	N/A			
Mean of drugs in positive screening test results (excluding alcohol)	2.72	N/A			

Note: N/A not assessed in general SUD group.

Note: P-values printed in bold indicate a statistically significant difference.

The presence of self-reported symptoms of depression and anxiety was similar in both groups (85.0% versus 84.2%). Psychotic symptoms, such as hallucinations (including drug-induced psychotic symptoms), were reported by 58.3% in the ADHD/SUD group and 64.2% in the general SUD group (ns).

A statistically significant difference was found in preferred substance abuse (*p* = .049). In the ADHD/SUD group, the stimulant drugs amphetamines and cocaine were preferred in 53.3%, compared with 30.8% in the general SUD group. A difference was also found in alcohol and benzodiazepine abuse: 6.7% and 3.3%, respectively, in the ADHD/SUD group reported these drugs as primary ones, while the percentages in the general SUD group were 21.7% and 9.0%. Only minor differences were found in abuse of other drugs. No data on drug screening at admission were available in the general SUD group, but drug screening data for the ADHD/SUD group showed that poly-drug abuse was common.

### 4. Retrospective report of ADHD symptoms during childhood and current cognitive ability

Retrospective self-ratings of ADHD symptoms during childhood and cognitive functions in the ADHD/SUD group (n = 60) were compared with those of the group of men with ADHD from an outpatient psychiatric setting (ADHD/Psych group, n = 107).

#### ADHD symptoms during childhood

Participants in the ADHD/SUD group reported significantly more ADHD symptoms in childhood as measured with WURS-25 (M = 60.15, SD = 17.27) compared to the ADHD/Psych group (M = 49.09, SD = 19.25) (*t* [120] = 3.21, *p* = .002, Cohen’s *d* = .62).

#### Cognition

**Figure 2:**
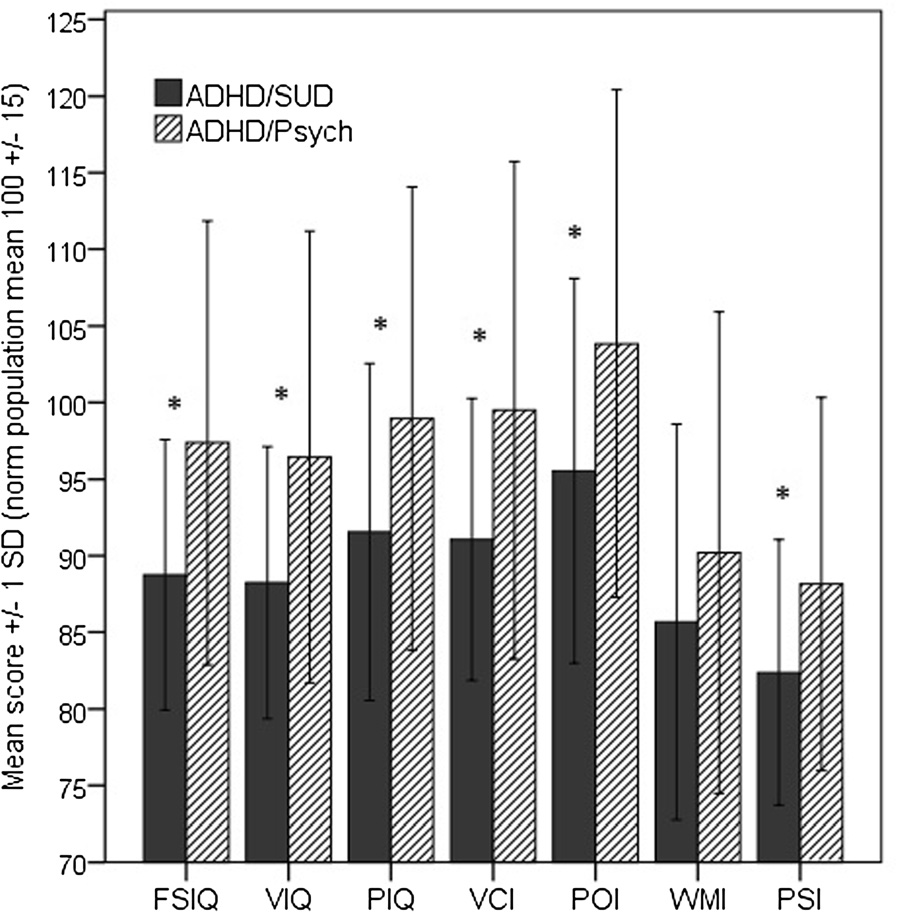
Cognitive ability in the ADHD/SUD group (n = 51, missing data n = 9) and the ADHD/out-patients group (n = 87, missing data n = 20).

Figure 2 shows the means values (+/− 1 SD) for measures of cognitive capacity in the ADHD/SUD group and the ADHD/Psych group.

Compared to the ADHD/Psych group, the ADHD/SUD group was found to have significantly poorer results in the full-scale IQ (n = 87, ADHD/Psych, and n = 51, ADHD/SUD, *t* [135.68] = 4.78, *p* < .001, Cohen’s *d* = .79). Similarly, further analyses of verbal IQ and non-verbal/performance IQ showed poorer results for the ADHD/SUD group: verbal IQ (n = 86, ADHD/Psych, and n = 51, ADHD/SUD, *t* [134.58] = 4.62, *p* = < .001, Cohen’s *d* = .77), non-verbal/performance IQ (n = 86, ADHD/Psych, and n = 51, ADHD/SUD, *t* [129.96] = 3.54, *p* = .001, Cohen’s *d* = .60). Analyses on the level of the four indexes of the WAIS-III showed similar pattern of results, i.e. the ADHD/SUD group performed poorer in all indexes: verbal comprehension index, VCI (n = 86, ADHD/Psych, and n = 51, ADHD/SUD, *t* [135.00] = 4.20, *p* < .001, Cohen’s *d* = .70); perceptual organization index, POI (n = 84, ADHD/Psych, and n = 51, ADHD/SUD, *t* [127.60] = 3.70, *p* < .001, Cohen’s *d* = .63); working memory index, WMI (n = 83, ADHD/Psych, and n = 51, ADHD/SUD, *t* [122.16] = 2.29, *p* = .129, Cohen’s *d = .*40); processing speed index, PSI (n = 84, ADHD/Psych, and n = 47, ADHD/SUD, *t* [124.92] = 3.50, *p* = .002 , Cohen’s *d* = .60). On controlling for FSIQ in an ANCOVA, the group differences in the working memory index (WMI) and speed of processing index (PSI) no longer reached statistical significance (both *p*-values > .10), indicating that there was no specific effect on EF in the ADHD/SUD group.

## Discussions

To the best of our knowledge, this is the first study characterizing adult males with ADHD and severe SUD in compulsory care. When compared with a general SUD group in compulsory care without a known ADHD diagnosis, the ADHD/SUD group was characterized by early and persistent antisocial behaviours and different abuse patterns. When compared with an outpatient group of males with ADHD (ADHD/Psych group), the ADHD/SUD group showed a more severe childhood symptomatology and poorer general cognitive ability.

Parental divorce and other unfavourable growing-up conditions, such as substance abuse and psychiatric disorders in parents, were common in both the ADHD/SUD group and the general SUD group. However, early separation of parents was more than twice as frequent in the ADHD/SUD group. Adverse life events during childhood, such as parental psychiatric disorders and substance abuse, have previously been reported in both individuals with ADHD [7,8,15] and individuals with substance abuse without ADHD [16-18]. Thus, the findings in the present study are in line with these reports.

Both the ADHD/SUD group and the general SUD group had a low educational level and the two groups did not differ largely from each other. However, the ADHD/SUD group had been subjected to a significantly higher degree to compulsory care (LVU) in childhood, which always includes a compulsory 9-year primary school education. In addition to the individuals being subjected to compulsory care during childhood, 37.7% of the ADHD/SUD group had received special education in primary school. Thus, in total, 80% of the ADHD/SUD group had received support to cope with school demands. Remarkably, only 13 of the 60 individuals in the ADHD/SUD group (21.7%) had a previous ADHD diagnosis at admission to SiS Institution Hornö.

Large proportions of both the ADHD/SUD group and the general SUD group had limited work experience, which was to be expected, bearing in mind the severe substance use disorder in both groups. However, we also observed a statistical trend in the ADHD/SUD group including several individuals with less than six months of work experience.

Antisocial behaviour was a distinguishing characteristic of the ADHD/SUD group, which not only had been subjected to a higher degree of compulsory care in childhood, but also imprisonment during adulthood, as compared to the general SUD group in compulsory care. The frequent co-existence of ADHD and conduct disorder (CD), oppositional defiant disorder (ODD), and adult antisocial behaviour has been reported previously [19-22] and it has also been suggested that it is the combination of ADHD and aggressive behaviour and/or CD during childhood that contributes to the most increased risk for later antisocial behaviour rather than ADHD as such [23-26]. Furthermore, the combination of ADHD and conduct problems during childhood has been suggested to be regarded as a distinct disorder, corresponding to the ICD-10 distinction between hyperkinetic disorder and hyperkinetic CD [23,26] and, with this distinction, it seems that many in the ADHD/SUD group in the current study would fall into the latter category. Torok et al. (2012) also found that CD is the strongest predictor for developing severe drug abuse, defined as ‘early onset, greater poly-drug use and more frequent stimulant use’, (and to be distinguished from less severe drug abuse) while ADHD without CD does not entail an increased risk for more severity of substance abuse [27]. Taken together, the ADHD/SUD group in the current study showed characteristics typical for both early antisocial development and the development of severe drug abuse [27-32].

Psychiatric co-morbidity is common in adults with ADHD [8,15,33-35], as well as in adults with SUD without ADHD [36-39]. In the current study, psychiatric symptoms were common in both the ADHD/SUD group and the general SUD group and the findings indicate that externalizing and antisocial behaviour, rather than psychiatric symptoms, are more distinctive for the ADHD/SUD group than for the general SUD group.

In a study of longer-term prison inmates with ADHD, all of whom had a diagnosis of substance abuse disorder, more than 70% preferred psycho-stimulants [3]. It has also been found that frequent stimulant use was more common in severe drug abuse than in less severe drug abuse, which, in turn, was found to be associated with CD [27]. In the current study, 53.3% in the ADHD/SUD group preferred amphetamine and cocaine, compared to 30.8% in the general SUD group. An additional difference between the groups was that the general SUD group preferred alcohol and benzodiazepines about three times more than the ADHD/SUD group. However, it should be noted that this variable described self-reported drug abuse. The drug screenings showed that poly-substance abuse was common in the ADHD/SUD group. No drug screening data were available for the general SUD group.

The comparison between the ADHD/SUD group and the ADHD/Psych group indicated medium-to-large differences in all measured parameters. The retrospective self-rating symptoms of ADHD during childhood were significantly higher and the estimated IQ was significantly lower in the ADHD/SUD group than in the ADHD/Psych group. In previous studies, IQ on a group level has been found to be lower in ADHD individuals than in the general population [8,40,41]. In the present study, the mean of full-scale IQ in the ADHD/Psych group was at the level of the general population, whereas, in the ADHD/SUD group, the mean was in the lower normal zone. Similar differences were found in a previous study [3] where male long-term prison inmates with ADHD and SUD were compared with males with ADHD from an outpatient psychiatric clinic, i.e. the estimated IQ was significantly lower in the ADHD/prison group. Furthermore, among offenders with intellectual disabilities, ADHD has been found to be the most frequently recorded developmental disorder [42].

The negative impact of various types of substance abuse on memory and executive functions has been reported frequently [43-49] and it can be assumed that the often early-onset, long-standing and severe abuse in the ADHD/SUD group has had a negative impact on cognitive functions, especially executive functions. However, executive dysfunctions are known to be associated with ADHD also without co-existing SUD and we did not observe any specific effects in tests loading on EF, but rather a general low cognitive ability in the ADHD/SUD group.

Altogether, the findings suggest that undiagnosed ADHD, especially in combination with antisocial behaviour and poor cognitive functions, constitute a high risk for the development of an extensive clinical burden, which, in turn, is associated with high risks, such as accidents, drug overdoses, and mortality, as well as family burdens and high care costs for society [50-52].

In many respects, the ADHD/SUD group and the general SUD group, showed similar clinical impairments and did not differ in socio-demographic background which could implicate need of intensive clinical intervention in both groups to reduce their burden of harm. However, findings in the current study also indicate that clients with ADHD in combination with SUD at a group level differ in some essential respects, both from the general LVM population with SUD and the ADHD/Psych group. This may implicate different treatment strategies concerning pharmacological and non-pharmacological treatment of ADHD with diverse comorbidities. Moreover, even ADHD-specific treatments, such as structured behaviour therapy for adults with ADHD [53], probably need to be modified when treating patients with complex comorbid patterns.

### Limitations

The present study has some limitations that may have had some impact on the observed results. One limitation is that the number of undiagnosed ADHD patients in the general SUD group was unknown. Bearing in mind the high prevalence of ADHD in substance abusers [2], it is plausible that some in the general SUD group had an undiagnosed ADHD. Likewise, the true prevalence may have also been higher at the SiS Institution Hornö since the assessments were voluntary and conducted only after a request from the social worker or from the patient himself. One conclusion is that the differences between the ADHD/SUD group and the general SUD group may have been larger if undiagnosed ADHD in the general SUD group could have entirely been ruled out. Furthermore, even though we had information on early antisocial behaviour in the ADHD/SUD group, the lack of formal DSM CD diagnosis can be seen as a limitation.

## Conclusions

In summary, the ADHD/SUD group exhibited a substantial and complex symptomatology with severe substance abuse and lifetime psychosocial problems, including early and persistent antisocial behaviour, as well as psychiatric co-morbidity and poor general cognitive ability. According to the findings, a clinical implication is that the total clinical burden of adults with ADHD and co-existing SUD should be identified and considered in treatment planning in terms of risk factors, treatment content, and the context and duration of treatment, as well as the short and long-term treatment goals.

## Competing interest

The authors declare that they have no competing interest.

## Authors’ contributions

BBM, JJ and TH designed the study. BBM and TH performed the statistical analysis. TH drew the figures. All authors contributed to the interpretation of the results. BBM wrote the first draft, supervised by TH and JJ. All authors read and commented on the manuscript, as well as approved the final manuscript.

## Pre-publication history

The pre-publication history for this paper can be accessed here:

http://www.biomedcentral.com/1471-244X/13/336/prepub
